# Editorial: Climate Change in Mountainous Areas and Related Health Effects

**DOI:** 10.3389/fphys.2021.768112

**Published:** 2021-10-14

**Authors:** Robert T. Mallet, Martin Burtscher, Annalisa Cogo

**Affiliations:** ^1^Department of Physiology and Anatomy, University of North Texas Health Science Center, Fort Worth, TX, United States; ^2^Department of Sport Science, University of Innsbruck, Innsbruck, Austria; ^3^Center for Exercise and Sports Science, University of Ferrara, Ferrara, Italy; ^4^Institute for Rehabilitation of Pediatric Asthma, Misurina, Italy

**Keywords:** climate – impact of, communicable and non-communicable diseases, point - of - care, hypoxia, avalanche accident, winter (sport) tourism

## Impact Statement

Although climate change is affecting the entire globe, its effects are particularly acute in mountainous regions, where the warming climate, infiltration of disease vectors, altered snow- and rainfall and increased avalanche risk are adversely impacting agriculture, health of human residents and animals, medical care, tourism, culture, and socioeconomic foundations. This editorial introduces five contributions of leading experts addressing distinct aspects of climate change and its disproportionate impact on the world's mountainous regions.

The burning of fossil fuels and the attendant release of greenhouse gases is increasingly impacting the global environment, producing abrupt changes in climate exemplified by global warming and increased air pollution. Although climate change has a profound worldwide impact, its effects are particularly acute in mountainous regions. The many consequences of warming in mountainous regions include glacial melting, permafrost degradation, abbreviated snow season, diminished snowpack, and limited water availability for mountain dwellers and lowlanders alike. Warming of mountain environments may allow mosquitoes, ticks and other disease vectors to afflict inhabitants of higher elevations.

Worldwide, over 500 million people dwell above 1,500 m (Tremblay and Ainslie, 2021), and over 100 million lowlanders visit these regions annually. The effects of climate change on high-altitude residents vary not only with latitude and altitude, but with cultural, socio-economic, and political factors, too. Available information on the health impact of climate change on mountain dwellers and visitors, and how these effects compare with those at lower elevations, is limited.

In this special issue, Dhimal et al. highlight the impact of climate change in the Hindu Kush Himalayan (HKH) region, which is warming more rapidly than the global average and has experienced increased precipitation over the last six decades. These phenomena are expected to affect key environmental and socioeconomic sectors including hydrology, agriculture, biodiversity, and human health. Climate change in the HKH region also is associated with increased prevalence of infectious and non-communicable diseases and malnutrition.

Another consequence of global warming is the decreased duration and accumulation of snowfall which can unfavorably affect both the tourism in mountainous regions and the possibility for residents to engage in winter sports. Frühauf et al. addressed this topic by comparing the effects of seeing images of scenarios affected vs. unaffected by climate change on anticipation and intention of performing recreational winter sports.

Strapazzon et al. discuss the potential effects of climate change on the frequency and characteristics of avalanches, demonstrating how changes in snow cover and land use have increased avalanche hazards and affected injuries and survival of persons trapped in avalanches.

Shao et al. present their study in a rat model of high altitude hypoxia demonstrating the impact of oxygen enrichment on pathophysiological responses to chronic hypoxia. Breathing oxygen-enriched air for 8 h/day mitigated the untoward effects of chronic hypoxia on the pulmonary circulation and blunted maladaptive right ventricular and pulmonary artery remodeling.

Finally, Nawrocki et al. describe their clinical field studies to validate the accuracy, utility, and reliability of portable blood gas analyzers to serve as essential point-of-care diagnostic tools in remote regions.

Knowledge of the effects of climate change on the health and disease of residents of and visitors to the world's mountainous regions is still deficient but is of the utmost clinical significance. We hope this Research Topic makes at least a modest contribution to the understanding of the complex interactions of climate, altitude, and human health, and stimulates researchers to conduct robust research on this topic.

## Author Contributions

RM wrote the first draft. AC and MB critically reviewed and all authors approved the final version of this editorial.

## Conflict of Interest

The authors declare that the research was conducted in the absence of any commercial or financial relationships that could be construed as a potential conflict of interest.

## Publisher's Note

All claims expressed in this article are solely those of the authors and do not necessarily represent those of their affiliated organizations, or those of the publisher, the editors and the reviewers. Any product that may be evaluated in this article, or claim that may be made by its manufacturer, is not guaranteed or endorsed by the publisher.
